# Dielectric Barrier Discharge Plasma Polymerization of N-Vinylimidazole: Structural Characterization and Cr^3+^ Coordination Behavior

**DOI:** 10.3390/polym18111332

**Published:** 2026-05-28

**Authors:** Nuri S. Ferguson, Hai-Feng Ji

**Affiliations:** Department of Chemistry, Drexel University, Philadelphia, PA 19104, USA; nsf44@drexel.edu

**Keywords:** atmospheric-pressure plasma, dielectric barrier discharge, plasma polymerization, N-vinylimidazole, poly(N-vinylimidazole), chromium(III) coordination

## Abstract

Plasma polymerization offers a solvent-free route to functional polymer materials, but the structural integrity and accessibility of functional groups in plasma-derived networks remain insufficiently validated. Herein, N-vinylimidazole (NVI) was polymerized using atmospheric-pressure dielectric barrier discharge (DBD) plasma in a liquid-film configuration to generate a chemically heterogeneous poly(N-vinylimidazole)-like material that could be recovered and evaluated in aqueous solution. ATR–FTIR and ^1^H NMR spectroscopy indicate substantial vinyl-group consumption with retention of imidazole functionality. Functional behavior was probed using chromium(III) (Cr^3+^) as a model metal ion. UV–Vis spectroscopy revealed systematic changes in the Cr^3+^ d–d transition region (~580–600 nm) with increasing polymer concentration, consistent with ligand-field perturbation arising from interactions with imidazole donor sites. A monotonic increase in absorbance with an increasing ligand-to-metal ratio was observed, followed by plateau behavior at higher ratios, indicating saturation of accessible coordination environments. These results demonstrate that plasma-polymerized material retains chemically accessible imidazole functionalities capable of coordinating transition-metal ions in solution, establishing atmospheric-pressure plasma polymerization as a viable route to functional imidazole-containing materials.

## 1. Introduction

Imidazole-containing polymers have attracted significant interest because the imidazole ring can participate in metal coordination, acid–base interactions, and hydrogen bonding, making these materials useful in adsorption, catalysis, sensing, and separation applications [1,2,3]. Among them, poly(N-vinylimidazole) (PVI) is particularly well studied due to its water compatibility and high density of nitrogen donor sites. Conventionally synthesized PVI, typically prepared by solution-phase radical polymerization, generally consists of relatively homogeneous linear chains and has demonstrated affinity toward a variety of metal ions through coordination and electrostatic interactions [4,5,6,7,8].

Plasma polymerization provides an alternative synthetic route to functional polymer materials. In contrast to conventional solution polymerization, plasma processes rely on energetic electrons, ions, radicals, and excited species that initiate fragmentation and recombination reactions under non-equilibrium conditions. As a result, plasma-polymerized materials often exhibit highly crosslinked and chemically heterogeneous structures [9,10,11]. Although this structural disorder can complicate characterization, plasma polymerization offers several advantages, including solvent-free processing, strong film adhesion, mild operating conditions, and compatibility with a wide range of substrates.

Previous studies involving N-vinylimidazole plasma polymerization have focused primarily on vapor-phase deposition of thin surface coatings, often using low-pressure systems to modify membrane or polymer interfaces [12]. These coatings are typically evaluated for surface charge, wettability, or interfacial performance rather than recovery of bulk material for direct chemical testing. Consequently, relatively little is known regarding whether imidazole functionalities remain chemically accessible after plasma polymerization, particularly in atmospheric-pressure systems where energetic discharge conditions may promote greater structural disorder.

Establishing functional-group retention is especially important for imidazole-containing plasma polymers because coordination behavior depends on the availability of ring nitrogen donor sites. If these functionalities remain intact and accessible, plasma polymerization could provide a convenient route to metal-binding materials without conventional solvent-based synthesis.

In the present study, N-vinylimidazole was polymerized using atmospheric-pressure dielectric barrier discharge (DBD) plasma in a liquid-film configuration under ambient conditions. The resulting material was recovered from the substrate and evaluated in aqueous solution, enabling direct study of intrinsic coordination behavior rather than only surface-bound effects. Polymer formation and chemical functionality were examined using ATR–FTIR and ^1^H NMR spectroscopy, while interaction with chromium(III) was investigated by UV–Vis spectroscopy as a model probe of accessible imidazole donor sites. These results demonstrate that atmospheric-pressure plasma polymerization can generate functionally active imidazole-containing polymer materials despite the structural heterogeneity inherent to plasma-derived networks.

## 2. Materials and Methods

### 2.1. Materials

N-Vinylimidazole (≥98.0%) was purchased from TCI Chemicals (Tokyo, Japan). Chromium(III) sulfate pentadecahydrate (Cr_2_(SO_4_)_3_·15H_2_O) was obtained from Cherokee Chemical Co. (Chicago, IL, USA). All reagents were used as received without further purification. Distilled water was used for the preparation of all aqueous solutions.

### 2.2. DBD Plasma Generation and Polymerization

**Figure 1:**
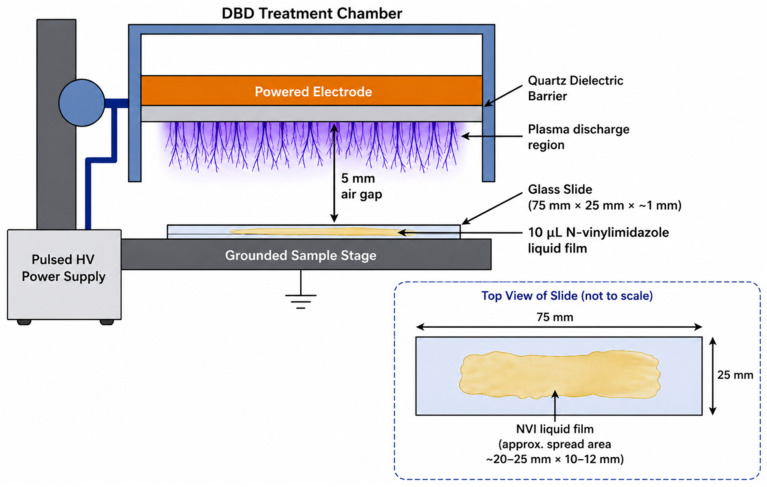
Schematic of the atmospheric-pressure dielectric barrier discharge (DBD) reactor used for N-vinylimidazole polymerization. A microsecond-pulsed high-voltage powered electrode separated from the grounded sample stage by a quartz dielectric barrier generated plasma within the 5 mm air gap above the sample surface. Neat N-vinylimidazole (10 µL) was deposited onto glass microscope slides (75 mm × 25 mm × ~1 mm), where it spontaneously spread into a thin liquid film prior to plasma exposure. The discharge in the gap is characteristic of atmospheric-pressure DBD operation and may consist of transient microdischarge filaments distributed across the treatment region [9,10,14].

Dielectric barrier discharge (DBD) plasma was generated using a microsecond-pulsed high-voltage power supply (FID Technology, Burbach, Germany) in an electrode–dielectric barrier configuration (Figure 1). Plasma treatments were conducted at a peak-to-peak voltage of 12 kV and a pulse repetition frequency of 2.5 kHz. Based on operating conditions and comparable systems reported in the literature, the input energy was estimated to be approximately 10 mJ per pulse [13]. The discharge gap between the lower surface of the quartz dielectric barrier and the sample surface was maintained at 5 mm throughout all experiments.

Standard glass microscope slides (75 mm × 25 mm × ~1 mm) were used as substrates. Prior to monomer deposition, slides were exposed to DBD plasma for 1 min to improve surface wettability and promote uniform spreading of the monomer film [9,10]. Following pre-treatment, 10 µL of neat N-vinylimidazole (NVI) was deposited onto each slide using a Gilson micropipette (Gilson, Inc., Middleton, WI, USA). The liquid spontaneously spread into a thin film covering an approximately rectangular/elliptical region of the slide surface (representative dimensions ~20–25 mm in length and ~10–12 mm in width).

The coated slides were then exposed to DBD plasma under ambient laboratory conditions for polymerization times of 3, 5, or 10 min, depending on the experiment. Unless otherwise noted, subsequent coordination studies were performed using the 5 min plasma-polymerized material. Three independently prepared 5 min samples were used for spectroscopic characterization (ATR–FTIR and pooled ^1^H NMR), while an additional set of ten independently prepared 5 min samples was used for gravimetric reproducibility measurements and preparation of pooled material for Cr^3+^ coordination studies.

After plasma exposure, samples were aged under ambient conditions for 72 h to allow post-plasma reactions and film stabilization. The resulting films appeared dry, adherent, and mechanically stable. Slides were weighed before and after treatment, and the net mass increase attributable to the deposited plasma-polymerized film was determined gravimetrically. For the 5 min treatment condition (*n* = 10), the recovered polymer mass was 7.64 ± 0.55 mg per slide (mean ± standard deviation), indicating good run-to-run reproducibility under the selected operating conditions. Preliminary gravimetric values for the 3 and 10 min treatment conditions were of the same general order of magnitude, with the 5 min condition selected for subsequent studies because it provided the best overall reproducibility. For solution-phase experiments requiring larger sample quantities, material recovered from multiple independently prepared slides was combined and used to prepare polymer stock solutions.

### 2.3. Spectroscopic Characterization

ATR–FTIR spectra were collected using a PerkinElmer Spectrum One FTIR spectrometer (PerkinElmer, Inc., Waltham, MA, USA) equipped with an attenuated total reflectance accessory. Spectra were acquired from 4000 to 650 cm^−1^ at 4 cm^−1^ resolution. Plasma-polymerized samples were analyzed after aging for 72 h under ambient conditions. Background spectra were collected in air prior to each measurement.

^1^H NMR spectra were acquired using a Shimadzu 500 MHz NMR spectrometer (Shimadzu Scientific Instruments, Columbia, MD, USA) with DMSO-d_6_ as the solvent. Unless otherwise noted, spectra shown correspond to the 5 min plasma-polymerized sample. Because the mass recovered from individual slides was limited, material from multiple independently prepared 5 min samples was combined to obtain sufficient signal intensity. Spectra were collected at ambient temperature using a standard one-dimensional single-pulse sequence (16 scans, 1 s relaxation delay). Chemical shifts were referenced to the residual solvent signal.

### 2.4. UV–Vis Analysis of Cr^3+^–Polymer Interactions

A stock Cr^3+^ solution (~4.0 × 10^−2^ mol L^−1^) was prepared by dissolving chromium(III) sulfate pentadecahydrate in distilled water. A stock solution of plasma-polymerized PVI was prepared by dissolving pooled 5 min plasma-polymerized material in distilled water to form a homogeneous aqueous stock solution. Polymer concentration (~9.7 × 10^−2^ mol L^−1^) was expressed on a repeat-unit equivalent basis using the molecular weight of the N-vinylimidazole repeat unit. Because plasma polymerization produces a distribution of fragment-derived structures rather than discrete polymer chains, a single well-defined molecular weight cannot be assigned. Accordingly, concentrations are reported on a repeat-unit equivalent basis [9,10,11].

Samples were prepared over ligand-to-metal ratios ([L]/[Cr^3+^]) of 0–7 while maintaining a constant Cr^3+^ concentration of 1.0 × 10^−2^ mol L^−1^. Appropriate volumes of polymer and Cr^3+^ stock solutions were combined and diluted to a final volume of 2.00 mL. Solutions were mixed thoroughly and equilibrated at room temperature for 15 min prior to analysis.

UV–Vis spectra were collected over 190–1100 nm using an Agilent 8453 UV–visible spectrophotometer (Agilent Technologies, Santa Clara, CA, USA). Polymer-only control samples were prepared and analyzed under identical conditions. Spectra shown for visualization were lightly smoothed using a moving-average routine; all quantitative values were obtained from unsmoothed data.

For quantitative comparison, absorbance values averaged over 585–590 nm, corresponding to the Cr^3+^ visible d–d transition region, were plotted as a function of ligand-to-metal ratio.

Additional comparison experiments using unpolymerized N-vinylimidazole (NVI) were conducted under analogous conditions; however, these data were not used for quantitative analysis due to turbidity effects observed at intermediate ligand-to-metal ratios.

## 3. Results and Discussion

### 3.1. ATR–FTIR Analysis

**Figure 2:**
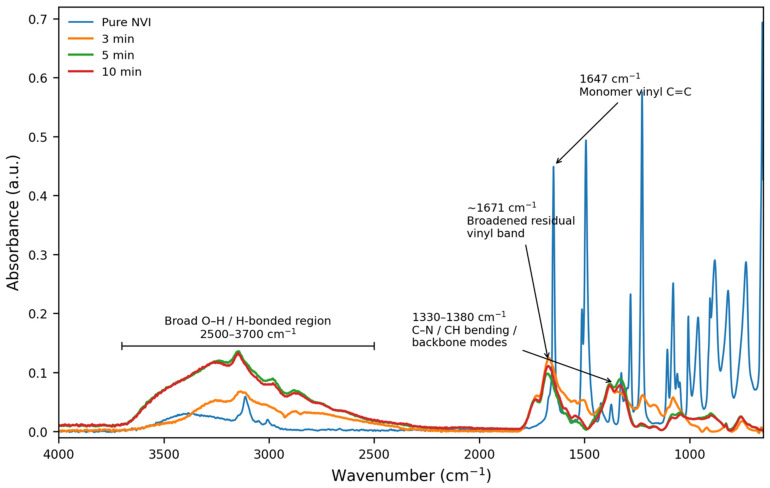
ATR–FTIR spectra of neat N-vinylimidazole (NVI) and NVI after 3, 5, and 10 min of dielectric barrier discharge (DBD) plasma exposure. The monomer C=C stretching band at ~1647 cm^−1^ is strongly attenuated after plasma treatment, while a weaker broadened residual vinyl-associated feature appears near ~1671 cm^−1^. Plasma-treated samples also display broad O–H stretching absorption (2500–3700 cm^−1^) and broadened features in the 1330–1380 cm^−1^ region attributed to overlapping C–N stretching, CH bending, and polymer-backbone vibrations. The similarity of the 5 and 10 min spectra suggests that substantial vinyl consumption occurs early during treatment, whereas longer exposure primarily promotes secondary network evolution.

ATR–FTIR spectra of neat N-vinylimidazole (NVI) and samples subjected to 3, 5, and 10 min of dielectric barrier discharge (DBD) plasma exposure are shown in Figure 2. The neat monomer exhibits a distinct absorption at approximately 1647 cm^−1^, assigned to the vinyl C=C stretching vibration. Following plasma treatment, this band decreases markedly in intensity, indicating consumption of vinyl functionality during plasma-initiated polymerization.

After 3 min of plasma exposure, the C=C band is already substantially attenuated relative to the monomer, suggesting that significant conversion occurs during the early stages of treatment. Concurrently, several sharp monomer bands in the fingerprint region become broadened or diminished, consistent with loss of monomer order and formation of a chemically heterogeneous polymeric network [9,10,11].

Broad absorption extending from approximately 2500 to 3700 cm^−1^ is observed in all plasma-treated samples and is assigned primarily to O–H stretching from adsorbed moisture, hydrogen-bonded hydroxyl groups formed during plasma exposure, and/or residual surface-bound water [9,10]. The greater prominence of this feature in plasma-treated samples is consistent with increased surface polarity and hydrogen-bonding capacity following plasma processing.

Additional broad absorptions emerge in the 1330–1380 cm^−1^ region, with maxima near 1331 and 1377 cm^−1^. These bands are reasonably assigned to overlapping C–N stretching vibrations of substituted imidazole environments, CH bending modes, and backbone-associated vibrations generated after conversion of the vinyl group into a more saturated, polymer-like framework [6,9,10]. Their broad character is consistent with a distribution of local bonding environments rather than a single well-defined structure.

In addition to decreased intensity, the residual vinyl-associated absorption becomes broader and shifts slightly toward higher wavenumber, from ~1647 cm^−1^ in the monomer to ~1671 cm^−1^ after plasma treatment. This behavior is consistent with a distribution of local bonding environments and possible residual unsaturation within the chemically heterogeneous plasma-derived network. Similar peak broadening and positional shifts have been reported previously for plasma-polymerized films [9,10,15].

Extending the plasma exposure from 3 to 5 and 10 min does not produce a clearly monotonic further decrease in the residual vinyl-associated feature, and the spectra obtained after 5 and 10 min remain broadly similar in the vinyl region. Instead, the more noticeable changes at longer treatment times are increased band broadening and redistribution of absorption intensity, particularly within the fingerprint region and the broad 2500–3700 cm^−1^ envelope. These observations suggest that much of the readily accessible vinyl functionality is consumed during the early stage of plasma exposure, while longer treatments primarily promote secondary network evolution, such as additional network formation, bond rearrangement, and oxidation, rather than extensive additional vinyl conversion [9,10,11]. Accordingly, the 5 min condition was selected for subsequent coordination studies as a practical intermediate treatment condition that provides substantial polymerization beyond the 3 min treatment while avoiding the greater structural modification associated with 10 min exposure.

Relative to conventionally synthesized poly(N-vinylimidazole), the plasma-polymerized material exhibits broader and less resolved vibrational features, reflecting the non-equilibrium and structurally heterogeneous nature of plasma polymerization [6]. Nevertheless, the persistence of absorptions attributable to imidazole-containing environments indicates that nitrogen-containing functionality is retained after treatment [9,11].

Collectively, the ATR–FTIR results support rapid plasma-induced polymerization of N-vinylimidazole with substantial vinyl consumption occurring during the early stages of treatment, followed by progressive network evolution at longer exposure times.

### 3.2. ^1^H NMR Characterization

**Figure 3:**
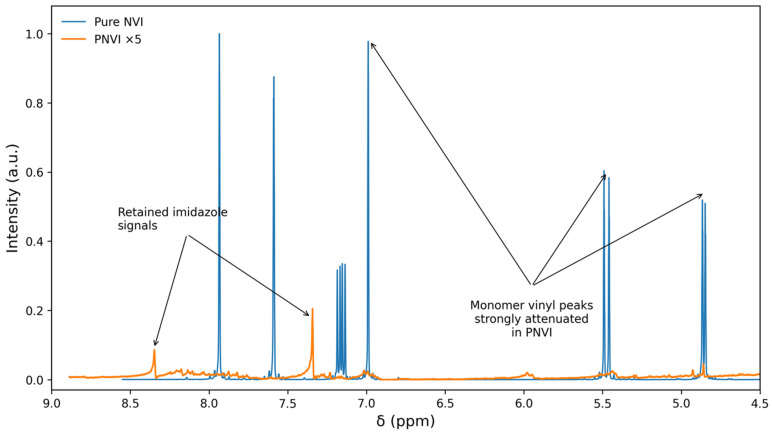
Overlay ^1^H NMR spectra (500 MHz, DMSO-d_6_) of neat N-vinylimidazole (NVI) and pooled independently prepared 5 min plasma-polymerized poly(N-vinylimidazole)-like material over the δ = 9.0–4.5 ppm region. Vinyl proton resonances present in the monomer at ~6.98, 5.50, and 4.85 ppm are strongly attenuated and no longer resolved after plasma treatment, consistent with consumption of vinyl functionality during polymerization. Broad retained resonances near ~8.35 and ~7.35 ppm are assigned to imidazole ring protons, indicating persistence of nitrogen-containing functionality. The plasma-polymerized spectrum was vertically scaled (×5) to facilitate visualization of low-intensity broad signals; intensities are therefore qualitative.

The ^1^H NMR spectrum of the plasma-polymerized material provides further evidence for polymer formation while reflecting the chemically heterogeneous nature of the resulting network (Figure 3). Spectra shown correspond to pooled independently prepared 5 min plasma-polymerized samples acquired in DMSO-d_6_.

In the imidazole proton region (8.6–7.1 ppm), two broad resonances are observed for the plasma-polymerized material, centered at approximately 8.35 ppm and 7.35 ppm. These signals are assigned to the C2–H proton (1H) and overlapping C4/C5–H protons (2H) of the imidazole ring, respectively. Relative to the sharp, well-resolved monomer resonances and spectra reported for conventionally synthesized poly(N-vinylimidazole), the pronounced broadening and loss of fine splitting observed here are consistent with chemical-shift dispersion arising from heterogeneous local environments within the plasma-derived polymer network [4,9,10].

In neat N-vinylimidazole, vinyl proton resonances are observed at approximately 6.98, 5.50, and 4.85 ppm. These signals are substantially attenuated and no longer resolved in the plasma-polymerized sample, indicating extensive consumption of vinyl functionality during plasma treatment. No resolved vinyl resonances attributable to residual monomer are observed within the sensitivity limits of the measurement.

Resonances corresponding to polymer-backbone CH and CH_2_ environments would be expected in the 1–4 ppm region; however, these signals are largely obscured by intense DMSO-d_6_ and residual water resonances and were therefore not analyzed in detail.

Overall, the retained imidazole resonances, disappearance of resolved vinyl signals, and broad line shapes are consistent with formation of a plasma-derived poly(N-vinylimidazole)-like material exhibiting significant structural disorder, a characteristic feature of polymers formed under dielectric barrier discharge plasma conditions.

### 3.3. Interaction of Plasma-Polymerized PVI with Cr^3+^ in Solution

#### 3.3.1. Spectroscopic Evidence of Cr(III)—Polymer Interaction

The functional activity of the plasma-polymerized material was evaluated through interaction with Cr^3+^ in aqueous solution using UV–Vis spectroscopy. All coordination experiments employed the pooled 5 min plasma-polymerized PVI-like material selected from the spectroscopic polymerization study. Because the plasma-polymerized material dissolved upon immersion in water, the interaction was examined under homogeneous solution-phase conditions rather than as a conventional surface adsorption process. Although plasma polymerization is often associated with highly crosslinked, insoluble networks, the present liquid-film configuration produces a chemically heterogeneous material that is soluble under the present experimental conditions, rather than a fully crosslinked, insoluble bulk network. Under these conditions, the observed spectral changes are attributed to coordination between Cr^3+^ and imidazole donor sites within the dissolved material.

A comparison experiment using unpolymerized N-vinylimidazole (NVI) was also performed under analogous conditions. At intermediate ligand-to-metal ratios, NVI solutions became visibly turbid, and the corresponding UV–Vis spectra exhibited significant baseline distortion consistent with light scattering effects. These conditions prevented reliable spectroscopic analysis of monomer-based systems. In contrast, plasma-polymerized samples remained optically clear and exhibited smooth, concentration-dependent spectral changes, supporting the suitability of the polymerized material for homogeneous solution-phase coordination analysis.

**Figure 4:**
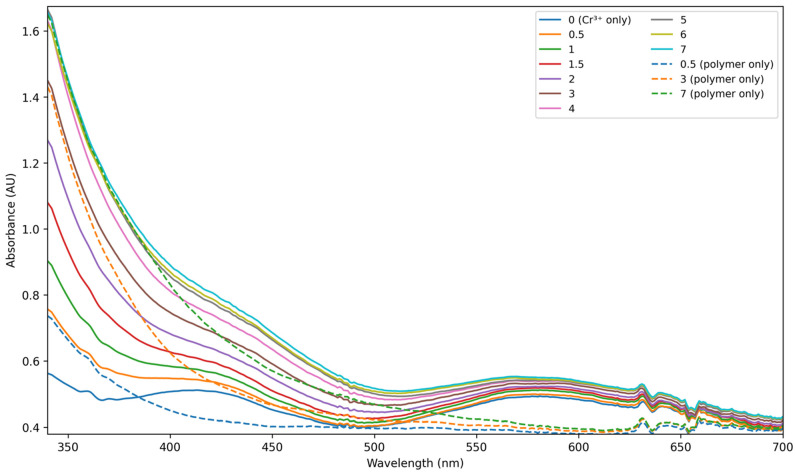
UV–Vis absorption spectra of Cr^3+^ (0.010 M) in aqueous solution containing increasing concentrations of pooled 5 min plasma-polymerized PVI, expressed as ligand-to-metal ratio ([L]/[Cr^3+^] = 0.5–7), recorded over the wavelength range of 340–700 nm. Progressive spectral changes are observed with increasing polymer concentration, including a systematic increase in the Cr^3+^ absorption band near 580–600 nm. Polymer-only controls (dashed lines) show negligible absorbance in this region, indicating that the observed increase arises primarily from Cr^3+^–polymer interaction rather than polymer background absorption. Spectra were lightly smoothed for visualization; quantitative analysis was performed using unsmoothed data.

UV–Vis absorption spectra of Cr^3+^ solutions containing increasing concentrations of plasma-polymerized PVI are shown in Figure 4. Although changes are observed across a broader wavelength range, the feature near 430 nm was not used for quantitative interpretation because it lies on a rising baseline and is more susceptible to overlap effects. In contrast, the visible-region band near 580–600 nm provides a more reliable basis for analysis because it exhibits systematic concentration-dependent changes, while polymer-only controls show negligible absorbance in this region.

Cr^3+^ exhibits broad d–d absorption in the visible region [16,17,18]. Upon increasing the ligand-to-metal ratio ([L]/[Cr^3+^]), a monotonic increase in absorbance is observed near 580–600 nm. This behavior is consistent with progressive modification of the chromium(III) ligand-field environment as imidazole nitrogen donors from the polymer interact with the metal center, in agreement with established coordination behavior of imidazole-containing ligands toward transition-metal ions [16,17,18]. Polymer-only control samples do not display the same trend, confirming that the observed spectral response arises from the Cr^3+^–polymer system rather than the polymer alone.

These results indicate that nitrogen-containing functionalities remain chemically accessible after plasma polymerization despite the structurally heterogeneous nature of the material.

#### 3.3.2. Effect of Polymer-to-Metal Ratio

To further evaluate coordination behavior, solutions were prepared over a range of polymer repeat-unit to Cr^3+^ molar ratios. Increasing polymer concentration produced progressive spectral changes, indicating an increasing extent of Cr^3+^–polymer interaction as more imidazole donor sites became available. This trend is consistent with coordination of Cr^3+^ by nitrogen-containing ligands and supports assignment of the observed spectral changes to Cr^3+^–imidazole interactions and is less consistent with nonspecific solution effects [17].

**Figure 5:**
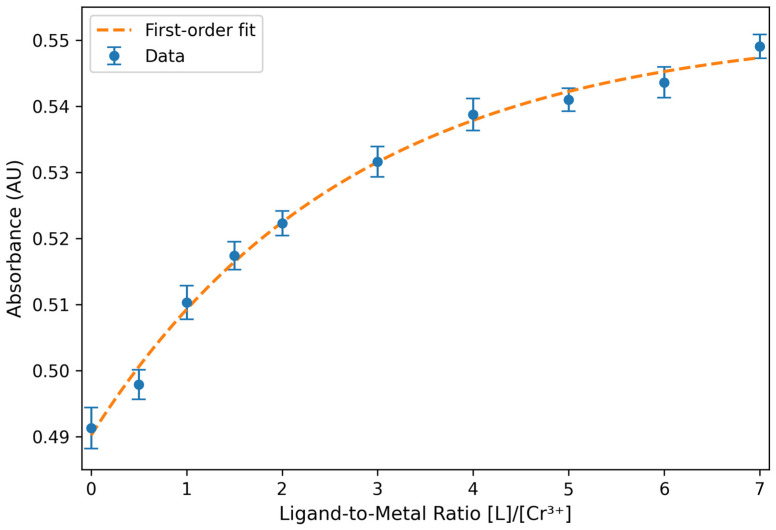
Absorbance of Cr^3+^ (0.010 M) in the presence of plasma-polymerized PVI as a function of ligand-to-metal ratio ([L]/[Cr^3+^] = 0–7), determined from the mean absorbance over the 585–590 nm region. Error bars represent ±1 standard deviation of absorbance values within the averaging window. A monotonic increase in absorbance is observed with increasing ligand ratio, approaching plateau behavior at higher [L]/[Cr^3+^]. The dashed line represents a first-order saturation fit used to guide the observed trend.

For quantitative comparison, absorbance values averaged over 585–590 nm were plotted as a function of the ligand-to-metal ratio (Figure 5). A monotonic increase in absorbance is observed with increasing polymer concentration, followed by an approach to plateau behavior at higher ratios (≥4). This trend indicates diminishing incremental spectral change once a substantial fraction of accessible coordination environments has been perturbed.

An empirical first-order saturation expression was applied to visualize the observed trend:

$$A=A_{\infty }(1-e^{-kL})+A_{0}$$

where A is the observed absorbance, $A_{\infty }$ represents the limiting absorbance at a high ligand-to-metal ratio, k is an empirical constant describing the extent to which saturation is approached, L is the ligand-to-metal ratio, and $A_{0}$ is the baseline absorbance.

Fitting yielded:

$$A=0.5514-0.0624e^{-0.3787L}$$

corresponding to a limiting absorbance of approximately 0.551.

Although the chemically heterogeneous plasma-derived network does not permit assignment of a single discrete stoichiometric complex, the saturation-like behavior is consistent with progressive occupation of a finite number of accessible imidazole coordination environments. This behavior is also consistent with perturbation or partial replacement of the original hydration sphere of Cr^3+^ by imidazole donor groups, as expected for ligand substitution processes in transition-metal complexes [16,17]. Because the polymer is dissolved under the experimental conditions, these results are most appropriately interpreted as a homogeneous coordination study rather than adsorption.

#### 3.3.3. Implications for Functional Group Retention in Plasma-Polymerized PVI

The present results demonstrate that plasma-polymerized PVI retains chemically accessible imidazole functionalities capable of coordinating transition-metal ions in aqueous solution. This finding is significant because plasma polymerization is often associated with highly crosslinked and structurally heterogeneous networks formed through fragmentation and recombination processes, which can potentially reduce functional-group accessibility.

Despite this structural disorder, the observed Cr^3+^–dependent spectral changes indicate that nitrogen donor sites remain functionally active after plasma treatment. These results therefore establish a clear link between spectroscopic evidence of retained imidazole chemistry and measurable coordination behavior in solution.

Unlike conventional linear or porous PVI-based materials, where metal-binding behavior may be strongly influenced by swelling, porosity, or mass-transport limitations, the present system reflects coordination interactions occurring within a dissolved chemically heterogeneous polymer network. Accordingly, these findings provide proof-of-concept that plasma-polymerized imidazole-containing materials can retain useful ligand functionality without requiring highly controlled polymer architectures or supporting matrices.

## 4. Conclusions

The combined spectroscopic and chromium-interaction results demonstrate that atmospheric-pressure dielectric barrier discharge (DBD) plasma polymerization of N-vinylimidazole produces a chemically heterogeneous yet functionally active poly(N-vinylimidazole)-like material. Although the plasma-derived, chemically heterogeneous network exhibits broadened and less resolved spectroscopic features relative to conventionally synthesized materials, ATR–FTIR and ^1^H NMR provide clear evidence of substantial vinyl-group consumption together with retention of imidazole functionality.

UV–Vis studies of Cr^3+^ solutions containing the plasma-polymerized material show systematic concentration-dependent changes in the visible d–d transition region, consistent with coordination-induced ligand-field perturbation arising from interaction with imidazole donor sites. The increase in absorbance with an increasing ligand-to-metal ratio, followed by plateau behavior at higher ratios, indicates that these retained functionalities remain chemically accessible in aqueous solution.

Together, these findings establish a clear structure–function relationship between retained imidazole chemistry and measurable metal-ion coordination behavior in a plasma-derived polymer network. More broadly, this work demonstrates that atmospheric-pressure DBD plasma polymerization is a viable route to functional nitrogen-containing materials and provides a foundation for future efforts aimed at improving material stability and optimizing coordination-driven aqueous-phase performance.

## Data Availability

The original contributions presented in this study are included in the article. Further inquiries can be directed to the corresponding author.
